# Performance of Artificial Intelligence Imaging Models in Detecting Dermatological Manifestations in Higher Fitzpatrick Skin Color Classifications

**DOI:** 10.2196/31697

**Published:** 2021-10-12

**Authors:** Pushkar Aggarwal

**Affiliations:** 1 College of Medicine University of Cincinnati Cincinnati, OH United States

**Keywords:** deep learning, melanoma, basal cell carcinoma, skin of color, image recognition, dermatology, disease, convolutional neural network, specificity, prediction, artificial intelligence, skin color, skin tone

## Abstract

**Background:**

The performance of deep-learning image recognition models is below par when applied to images with Fitzpatrick classification skin types 4 and 5.

**Objective:**

The objective of this research was to assess whether image recognition models perform differently when differentiating between dermatological diseases in individuals with darker skin color (Fitzpatrick skin types 4 and 5) than when differentiating between the same dermatological diseases in Caucasians (Fitzpatrick skin types 1, 2, and 3) when both models are trained on the same number of images.

**Methods:**

Two image recognition models were trained, validated, and tested. The goal of each model was to differentiate between melanoma and basal cell carcinoma. Open-source images of melanoma and basal cell carcinoma were acquired from the Hellenic Dermatological Atlas, the Dermatology Atlas, the Interactive Dermatology Atlas, and DermNet NZ.

**Results:**

The image recognition models trained and validated on images with light skin color had higher sensitivity, specificity, positive predictive value, negative predictive value, and F1 score than the image recognition models trained and validated on images of skin of color for differentiation between melanoma and basal cell carcinoma.

**Conclusions:**

A higher number of images of dermatological diseases in individuals with darker skin color than images of dermatological diseases in individuals with light skin color would need to be gathered for artificial intelligence models to perform equally well.

## Introduction

### Background

In dermatology, artificial intelligence (AI) is poised to improve the efficiency and accuracy of traditional diagnostic approaches, including visual examination, skin biopsy, and histopathologic examination [1]. Deep-learning image recognition models have had success in differentiating between dermatological diseases using images of light-skinned individuals. However, when these models are tested on images of people with skin of color, the performance drops [2]. It is thought that the primary reason for this difference is the lack of available images of dermatological diseases in individuals with darker skin color (Fitzpatrick classification of skin types 4 and 5) [3]. However, is it also possible that even when the same number of images are available, image recognition models will have a harder time differentiating between dermatological diseases in individuals with Fitzpatrick skin types 4 and 5 compared to skin types 1, 2, and 3?

### Objective

The objective of this research was to assess whether image recognition models perform differently when differentiating between dermatological diseases in individuals of color (Fitzpatrick skin types 4 and 5) than when differentiating between the same dermatological diseases in Caucasians (Fitzpatrick skin types 1, 2, and 3) when both models are trained on an equal number of images.

## Methods

Open-source images of melanoma and basal cell carcinoma (BCC) were acquired from the Hellenic Dermatological Atlas [4], the Dermatology Atlas [5], the Interactive Dermatology Atlas [6], and DermNet NZ [7]. Two image recognition models were trained, validated, and tested using methodology as described previously [8]. TensorFlow [9], an open-source software library by Google, was used as a deep-learning framework and was used to retrain Inception, version 3 (v3). Inception v3 is a deep convolutional neural network. This neural network consists of a hierarchy of multiple computational layers that each have an input and output. All layers except the final layer of this neural network are pretrained with more than 1.2 million images. The final layer of the neural network was retrained with the gathered dermatological images. During the retraining process, the neural network underwent both a training and validation step. In the training step, the inputted images were used to train the neural network. In the validation step, inputted naïve images were used to iteratively assess training accuracy [10].

After the model had been retrained (trained and validated), a user-inputted testing/assessment step was performed in which test images were inputted and the results were statistically analyzed. The program assessment output is expressed in terms of percentages of the probability of each of the dermatological manifestations for each testing image inputted. R software (R Foundation for Statistical Computing) [11] was used to perform the statistical analysis. Sensitivity, specificity, positive predictive value (PPV), negative predictive value (NPV), and F1 score were calculated for each dermatological manifestation. The F1 score is the harmonic average of the sensitivity and PPV (mean of the recall and precision).

The goal of each model was to differentiate between melanoma and BCC.

The first model was:

- Trained on 150 images of individuals with light skin color (Fitzpatrick skin types 1, 2, and 3), 75 melanoma and 75 BCC images;
- Validated on 38 images of individuals with light skin color (Fitzpatrick skin types 1, 2, and 3), 19 melanoma and 19 BCC images;
- Tested on 30 images of individuals with light skin color (Fitzpatrick skin types 1, 2, and 3), 15 melanoma and 15 BCC images.

The second model was:

- Trained on 150 images of individuals with skin of color (Fitzpatrick skin types 4 and 5), 75 melanoma and 75 BCC images;
- Validated on 38 images of individuals with skin of color (Fitzpatrick skin types 4 and 5), 19 melanoma and 19 BCC images;
- Tested on 30 images of individuals with skin of color (Fitzpatrick skin types 4 and 5), 15 melanoma and 15 BCC images.

Area under the receiver operating characteristic (AUC) curves for melanoma and BCC were calculated to determine the performance of the two models.

## Results

When asked to differentiate between melanoma and BCC, the image recognition model trained and validated on images of light skin color had higher sensitivity, specificity, PPV, NPV, and F1 score than the image recognition model trained and validated on images of skin of color (Table 1).

In predicting melanoma, the image recognition model trained and validated on images of light skin color had a sensitivity of 0.60, specificity of 0.53, PPV of 0.56, NPV of 0.57, and F1 score of 0.58. On the other hand, in predicting melanoma, the same image recognition model trained and validated on images of skin of color had a sensitivity of 0.53, specificity of 0.47, PPV of 0.50, NPV of 0.50, and F1 score of 0.52.

In predicting BCC, the image recognition model trained and validated on images of light skin color had a sensitivity of 0.53, specificity of 0.60, PPV of 0.57, NPV of 0.56, and F1 score of 0.55. On the other hand, for prediction of BCC, the same image recognition model trained and validated on images of skin of color had a sensitivity of 0.47, specificity of 0.53, PPV of 0.50, NPV of 0.50, and F1 score of 0.48.

**Figure 1:**
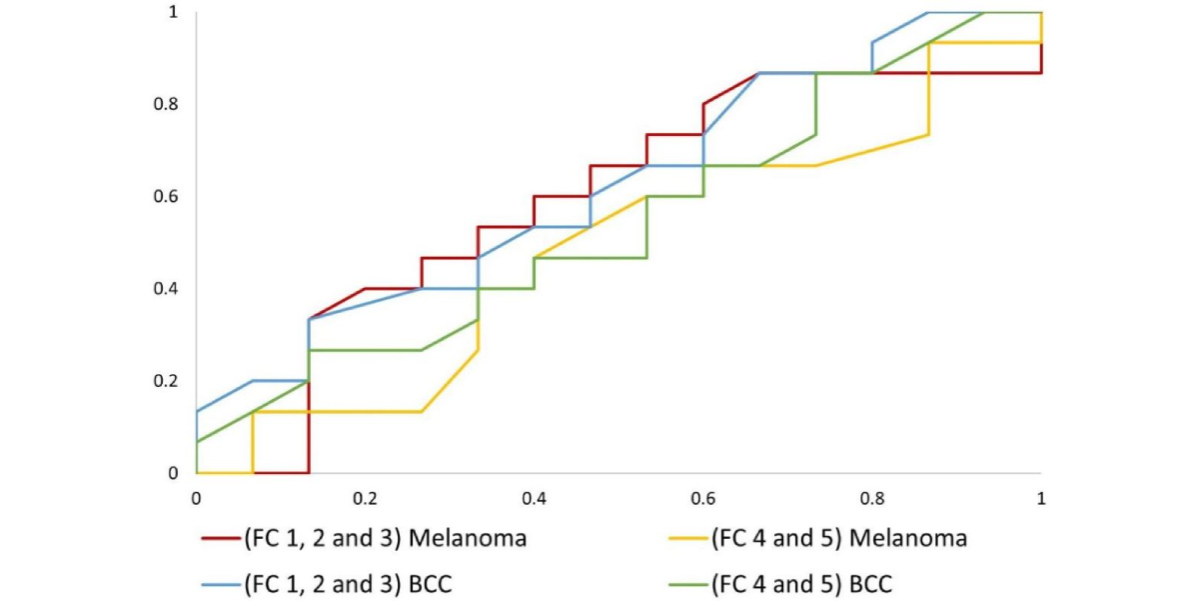
Receiver operating characteristic curves for melanoma and basal cell carcinoma (BCC) in each of the two models for different skin types. FC: Fitzpatrick classification.

The average AUC for the two light skin color image recognition models was 0.598, compared to 0.500 (values point out the difference) for the skin of color image recognition models (Table 1 and Figure 1).

**Table 1:** Statistical measures of the deep-learning model trained, validated, and tested on different Fitzpatrick skin type classifications (types 1, 2, and 3 vs types 4 and 5) for evaluating melanoma and basal cell carcinoma.

Measure	Melanoma model		Basal cell carcinoma model	
	Skin types 1, 2, and 3	Skin types 4 and 5	Skin types 1, 2, and 3	Skin types 4 and 5
Sensitivity	0.60	0.53	0.53	0.47
Specificity	0.53	0.47	0.60	0.53
Positive predictive value	0.56	0.50	0.57	0.50
Negative predictive value	0.57	0.50	0.56	0.50
F1 score	0.58	0.52	0.55	0.48
Area under the receiver operating characteristic curve	0.59	0.57	0.60	0.53

## Discussion

### Limitations

The number of images available was limited for Fitzpatrick skin types 4 and 5; as such, both the light skin color and skin of color models were investigated with this constraint for the number of images used during training. A larger sample size would have been better to test if the results recur consistently.

### Conclusion

When the same number of images is used for training, validation, and testing, the AI model that was provided images of melanoma and BCC belonging to Fitzpatrick classification skin types 1, 2, and 3 performed better than the AI model that was provided with images of melanoma and BCC in skin types 4 and 5. This may be because dermatological diseases can have more variability in presentation in individuals with darker skin; additionally, cutaneous manifestations may not be as easily distinguished from the surrounding skin in darker-skinned individuals. As such, a higher number of images of skin of color with dermatological diseases than images of light skin color with dermatological diseases would need to be gathered for the AI models to perform equally well.

## Abbreviations

AI: artificial intelligence
AUC: area under the receiver operating characteristic
BCC: basal cell carcinoma
NPV: negative predictive value
PPV: positive predictive value
